# Effects of inorganic and organic amendment on soil chemical properties, enzyme activities, microbial community and soil quality in yellow clayey soil

**DOI:** 10.1371/journal.pone.0172767

**Published:** 2017-03-06

**Authors:** Zhanjun Liu, Qinlei Rong, Wei Zhou, Guoqing Liang

**Affiliations:** 1 College of Natural Resources and Environment, Northwest A & F University, Yangling, China; 2 Ministry of Agriculture Key Laboratory of Crop Nutrition and Fertilization, Institute of Agricultural Resources and Regional Planning, Chinese Academy of Agricultural Sciences, Beijing, China; RMIT University, AUSTRALIA

## Abstract

Understanding the effects of external organic and inorganic components on soil fertility and quality is essential for improving low-yielding soils. We conducted a field study over two consecutive rice growing seasons to investigate the effect of applying chemical fertilizer (NPK), NPK plus green manure (NPKG), NPK plus pig manure (NPKM), and NPK plus straw (NPKS) on the soil nutrient status, enzyme activities involved in C, N, P, and S cycling, microbial community and rice yields of yellow clayey soil. Results showed that the fertilized treatments significantly improved rice yields over the first three experimental seasons. Compared with the NPK treatment, organic amendments produced more favorable effects on soil productivity. Notably, the NPKM treatment exhibited the highest levels of nutrient availability, microbial biomass carbon (MBC), activities of most enzymes and the microbial community. This resulted in the highest soil quality index (SQI) and rice yield, indicating better soil fertility and quality. Significant differences in enzyme activities and the microbial community were observed among the treatments, and redundancy analysis showed that MBC and available N were the key determinants affecting the soil enzyme activities and microbial community. The SQI score of the non-fertilized control (0.72) was comparable to that of the NPK (0.77), NPKG (0.81) and NPKS (0.79) treatments but significantly lower compared with NPKM (0.85). The significant correlation between rice yield and SQI suggests that SQI can be a useful to quantify soil quality changes caused by different agricultural management practices. The results indicate that application of NPK plus pig manure is the preferred option to enhance SOC accumulation, improve soil fertility and quality, and increase rice yield in yellow clayey soil.

## Introduction

Yellow clayey soil covers an area of 1.4 × 10^6^ hm^2^ in southern China [1]. Unfortunately, the low organic matter content and nutrient availability in this soil have resulted in low crop productivity [2]. Increasing demand for food supply or security in this region requires enhancing the soil quality to improve crop production.

Soil organic matter (SOM) is central to soil function and quality. Soil carbon (C) sequestration in agricultural soil has been suggested as a strategy to improve soil quality [3]. The amelioration of soil physicochemical properties by increasing soil organic carbon (SOC) has been proposed for increasing crop yields [4]. To maintain and improve SOC, management practices such as fertilization, no tillage, and rice straw incorporation have been applied to farming systems [5–7]. Chemical fertilization is not always useful for short-term enhancement of SOC [8]. In contrast, the combination of chemical fertilizer with organic materials showed great potential for SOC sequestration in the paddy soils of southern China [9], especially soils with low levels of organic matter [10]. Organic soil management can substantially improve soil structure [11], help retain C in the surface soil, and increase crop yields in rice-rice crop systems [12]. Exogenous applications of organic materials (e.g. green manure, farmyard manure, and straw) can reduce the amounts of chemical fertilizers used and compensate for soil C losses caused by land-use changes [13]. Thus, amending soil with organic materials is a promising strategy to build-up C levels in the paddy soils of subtropical China ([10, 14, 15]. Yuan *et al*. (2014) found that rice straw retention was effective for increasing SOC and improving soil fertility and productivity in yellow clayey soils [16]. However, these studies focused on the individual effects rather than combination effects, of specific organic amendments on soil physicochemical properties. The biological properties of soils have been the focus of recent studies [17–20]. Information about the differences in biological responses to chemical fertilizer and organic manure remains limited.

Soil quality information is important for developing appropriate anti-degradation measures and designing sustainable agricultural management practices [21]. Soil quality assessment through the development of a soil quality index (SQI) can indicate the positive or negative effects of field practices and integrate information from soil indicators into the management process [22]. Monitoring changes in soil quality indicators following a specific management strategy is a useful approach to determine the quality status of a soil [23]. However, most studies have focused on soil physicochemical characteristics alone while ignoring biological variables. Previous studies focused on measuring the productivity and have usually failed to develop a SQI, although a SQI may be more useful to measure the integrated effect of management strategies on soil quality and sustainability [2, 23]. Biological properties are the most sensitive indicators of changes in the soil quality of rice production systems due to their rapid responses to environmental changes [24]. Consequently, these biological properties might be particularly useful for characterizing soil fertility and quality changes in short-term experiments. A field experiment was conducted in yellow clayey soil with a double rice cropping system in subtropical China. Our objectives were to (i) examine the effects of inorganic and organic amendments on soil nutrient status, enzyme activities, and microbial communities, (ii) evaluate soil quality changes using a SQI, and (iii) identify viable options for improving the fertility and productivity of yellow clayey soil.

## Materials and methods

### Study area

This study began during the early-rice season in 2012 and was conducted in an agricultural field, located in Jingshan county, Hubei, China (30°51’N and 113°07’E, 35 m above the sea level). The field study was carried out on private land with the permission of the land owner, and did not involve endangered or protected species. The region has a subtropical monsoon climate with a mean annual temperature of 21.0°C, precipitation of 1,179 mm and evapotranspiration of 1,500 mm. The tested yellow clayey paddy soil is a Udalf soil with clay loam texture (USDA soil classification). The soil is developed from yellow sandstone and quaternary yellow clay, and the major clay minerals are hydrous mica and kaolinite. Based on farmers surveys, conventional fertilization focused on mineral fertilizers, and the fertilizer types were CO(NH_2_)_2_, Ca(H_2_PO_4_)_2_ and KCl for N, P, and K, respectively. Similar fertilization was employed for early rice and late rice. For each cropping season, we applied 180 kg N ha^−1^, 45 kg P_2_O_5_ ha^−1^ and 60 kg K_2_O ha^−1^. Coupled basic fertilizer and once-over tillage were conducted before each cropping season, and all P and K fertilizers during the early-rice and late-rice seasons were applied by basal dressing all in one time. Meanwhile, 40% of fertilizer N was applied as a basal fertilizer, 30% during the tillering stage and 30% during the booting stage. At the beginning of the experiment, the soil in the plough horizon (0–20 cm) had an initial bulk density of 1.23 Mg m^−3^, a pH of 6.3 (soil:water, 1:2.5), 11.57 g organic carbon kg^−1^, 0.74 g total nitrogen (N) kg^−1^, and 47.2 mg available N kg^−1^, 5.1 mg P_2_O_5_ kg^−1^ and 94.7 mg K_2_O kg^−1^, respectively. The study area had a double rice crop system with the early rice growth season from April to July and the late rice growth season from July to October.

### Experimental design

The experiment included five fertilization treatments: (1) unfertilized control (CK), (2) urea plus superphosphate and potassium chloride (NPK), (3) NPK + green manure (*Astragalus sinicus* L., 6.44% C, 0.38% N, 0.12% P_2_O_5_, 0.28% K_2_O) (NPKG), (4) NPK + pig manure compost (37.7% C, 2.98% N, 2.43% P_2_O_5_, 1.92% K_2_O) (NPKM), and (5) NPK + rice straw (48.3% C, 1.93% N, 0.22% P_2_O_5_, 3.24% K_2_O) (NPKS). The experimental design was a randomized complete block with four replicates or plots for each treatment. Each plot had an area of 20 m^2^ (4 m × 5 m) and was isolated by a 1 m depth of cement baffle plates.

Under the double rice growing system, the cultivar “Liangyou 287” was grown for the early season (15 April to 15 July) and the cultivar “Tyou 250” was grown for the late season (20 July to 30 October). The chemical fertilizer types and application rates used in this study were similar to those used by local farmers. For each rice season the same rates of 180 kg N ha^−1^, 90 kg P_2_O_5_ ha^−1^ and 120 kg K_2_O ha^−1^ were applied to all fertilization treatments. The amount of each type of organic material applied was determined according to its organic carbon content. Yellow clayey paddy soil has low productivity, and the estimated amount of rice straw incorporation is approximately 3000 kg ha^−1^ according to other studies [16, 25]. Thus, a total of 1,450 kg C ha^−1^ determined by the recommended rice straw incorporation was applied in each crop growth season in green manure, pig manure and rice straw treatment at the rates of 22,500 kg ha^−1^, 3,850 kg ha^−1^, and 3,000 kg ha^−1^, respectively. For each season, all chemical fertilizer phosphorus, potassium and organic materials were applied by basal dressing all in one time, while all chemical N were applied in three times (40% as basal, 30% of each at 30 and 45 days after transplanting). Ameliorants and basal fertilizer were evenly broadcast onto the soil surface and immediately incorporated into the plowed soil (0–20 cm depth) by tillage.

### Soil sampling and analysis

**Fig 1:**
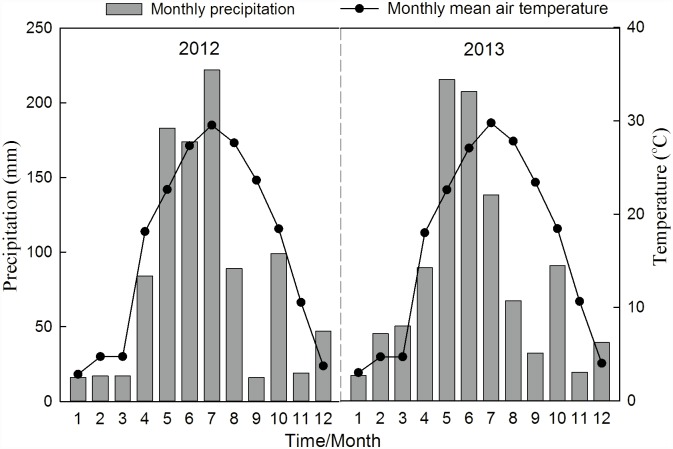
Monthly precipitation and mean air temperature over a two-year period (2012–2013).

To obtain more relatively significant changes in soil properties, we did not collect soil samples during the first trial season. Soil sampling in the plough horizon (0–20 cm depth) was conducted in the following 2-consecutive growing seasons, including the 2012 late rice season and the 2013 early rice season. Monthly precipitation and mean air temperatures from 2012 to 2013 are shown in Fig 1. In each plot, eight cores (4.0 cm diameter) were randomly sampled; the detailed sampling procedure is shown in S1 Fig. Half of each core soil was retained by vertically dividing it into two parts using a knife along the soil profile, and then mixed into one composite sample (~2000 g). Soil samples were kept on ice, immediately transported to the laboratory and then divided into three sub-samples. One sub-sample (~1000 g) was air-dried at 25°C for chemical analysis, one sub-sample (~500 g) was stored at 4°C for enzyme analysis and the third sub-sample (~500 g) was freeze-dried prior to be stored at −18°C for phospholipid fatty acid analyses (PLFA).

The concentrations of SOM (K_2_Cr_2_O_7_–external heating method), total nitrogen (TN; micro–Kjeldahl method), pH (soil:water, 1:2.5), available nitrogen (AN, alkalized nitrogen method), available phosphorus (AP, 0.5 M NaHCO_3_ extraction), and available potassium (AK, 1.0 M ammonium acetate extraction) were determined following the procedures described by Lu (2000) [26].

Microbial biomass C and N were analyzed using the chloroform fumigation-incubation method and determined as described by Vance *et al*. (1987) [27] and Shen *et al*. (1984) [28], respectively.

The activities (μmol h^−1^ g^−1^) of enzymes (see Table 1), except phenol oxidase and peroxidase, were assayed using the microplate fluorometric protocol following the procedure of Ai et al. (2015) [29]. The activities of phenol oxidase and peroxidase were colorimetrically measured in a clear 96-well microplate according to Zhang *et al*. (2015) [30].

**Table 1:** Enzymes with corresponding commission number (EC), corresponding substrate, and the abbreviation used in this study.

Enzyme	Abbreviation	Substrate	EC
Phosphomonosterase	Pho	4-MUB^a^-phosphate	3.1.3.1
Sulfatase	Sul	4-MUB-sulfate	3.1.6.1
β-Glucosidase	βG	4-MUB-β-D-glucoside	3.2.1.21
Cellobiohydrolase	CBH	4-MUB-β-D-cellobioside	3.2.1.91
N-Acetyl-glucosaminidase	NAG	4-MUB-N-acetyl-β-D-glucosaminide	3.2.1.30
α-Glucosidase	αG	4-MUB-α-D-glucoside	3.2.1.20
Phenol oxidase	PhOx	L-DOPA^b^	1.10.3.2
Peroxidase	Perox	L-DOPA	1.11.1.7

^a^ 4-MUB 4-methylumbelliferyl

^b^ L-DOPA L-3,4-dihydroxyphenylalanine

Microbial community structure was determined using PLFA analyses [31]. Total microbial mass was estimated using the total concentrations of PLFAs (nmol g^−1^). The abundance of individual PLFAs was their % mol abundance. The PLFAs were divided into various taxonomic groups based on previously published PLFA biomarker data [32]. We used fatty acids iC15:0, aC15:0, iC16:0, iC17:0 and aC17:0 as Gram-positive bacteria (G^+^) biomarkers; C16:1ω7c, C18:1ω7c and cyC17:0 as Gram-negative bacteria (G^−^) biomarkers; and the sum of G^+^, G^−^, together with C:15, C17:0 and cyC19:0ω11,12c as the total bacterial biomass. The sum of 10MeC16:0 and 10MeC18:0 was regarded as an indicator of actinomycetes. The fatty acids C18:2ω6.9c and C16:1ω5c were used as biomarkers for fungi and arbuscular mycorrhizal fungi (AMF), respectively.

### Developing a Soil Quality Index (SQI)

The total data set (TDS) method was used to develop a SQI, and all of the 22 determined variables were considered. Evaluation of soil quality was accomplished in three steps. First, a principal component analysis (PCA) was conducted using all observations of the measured soil properties, and the weight for each TDS indicator was calculated by its communality, which was equal to the ratio of its communality divided by the sum of the communalities of all TDS indicators [33]. Second, soil properties including SOM, TN, AN, AP and AK were normalized and scored using a standard scoring function, and the corresponding threshold values were used according to Qi *et al*. (2009) [34] and Li *et al*. (2013) [35]. For pH, a neutral value (7.0) was considered desirable and therefore it was taken as ‘higher is better’ because all values were less than 7. Because yellow clayey paddy soil is typically characterized by low productivity, the remaining biochemical and microbial properties without a certain threshold value were scored and normalized using the linear scoring function described by Liebig *et al*. (2001) [36]. For MBC, the highest value received a score of 1, and the other observations were divided by the highest value to normalize them. The same procedure was carried out for MBN, enzymatic activities and soil microbial groups. Third, the SQI was calculated using the following equation [37]:

$$SQI=\sum_{i=1}^{n}Wi\times Si$$ (1)

Where *Wi* is the assigned weight of each indicator, Si is the indicator score, and n is the number of variables.

### Data analysis

Data were statistically analyzed using SPSS18.0. One-way analysis of variance (ANOVA) was used to test all parameters, and the least significant difference (LSD) method at the probability level of 0.05 was used to separate the mean differences of the soil attributes. Redundancy analysis (RDA) with the Monte Carlo permutation test (499 permutations) was performed to determine if soil enzyme activity or community composition are correlated with soil properties, as implemented in Canoco for Windows version 4.5.

## Results

### Chemical properties

Soil pH, total N and SOM were not significantly affected by inorganic or organic amendments over the two selected rice seasons (Table 2). In contrast, soil available P and K differed significantly among the fertilization treatments. The concentrations of AP and AK were generally highest under NPKM and NPKS, respectively. Table 2 also shows that there were no significant differences for available N among the tested treatments until the third planting season (early rice, 2013), and the organic amendments (NPKG, NPKM and NPKS) resulted in equal AN contents. However, their values were significantly higher than those of the CK and NPK treatments.

**Table 2:** Variations in soil chemical properties under different fertilizations in 2012 and 2013.

Year	Treatment	pH	SOM (g/kg)	Total N (g/kg)	AN (mg/kg)	AP (mg/kg)	AK (mg/kg)
	CK	6.76±0.12a	18.94±1.27a	1.21±0.14a	79.19±7.94a	8.07±0.65b	107.33±2.74b
	NPK	6.74±0.10a	19.09±2.37a	1.23±0.15a	77.81±8.64a	8.02±1.86b	121.03±3.84ab
2012	NPKG	6.76±0.13a	18.63±3.92a	1.19±0.13a	90.89±7.77a	7.71±1.90b	120.72±7.11ab
	NPKM	6.86±0.07a	21.33±5.54a	1.25±0.20a	89.52±4.73a	11.87±0.52a	121.63±5.23ab
	NPKS	6.85±0.06a	22.06±2.06a	1.18±0.26a	90.89±6.28a	9.44±3.40ab	130.93±16.18a
	CK	6.89±0.13a	20.96±0.93a	1.35±0.06a	72.10±8.31b	11.74±1.23b	131.65±6.21b
	NPK	6.94±0.04a	21.80±2.14a	1.35±0.14a	77.63±7.26b	12.96±1.49ab	131.22±11.81b
2013	NPKG	6.96±0.07a	22.10±3.63a	1.38±0.20a	93.49±6.81a	13.57±3.04ab	159.12±6.54a
	NPKM	6.97±0.04a	23.86±3.10a	1.47±0.17a	90.13±3.90a	17.82±5.43a	157.81±10.97a
	NPKS	6.90±0.13a	24.89±4.02a	1.44±0.39a	91.09±3.63a	16.46±2.00ab	159.47±22.83a

Data are means ± SE, *n* = 3. Different letters indicate significant differences between fertilizations at *P*<0.05. Abbreviations: AK, available potassium; AN, available nitrogen; AP, available phosphorus; SOM, soil organic matter; TN, total nitrogen.

### Microbial biomass C/N and enzyme activities

**Fig 2:**
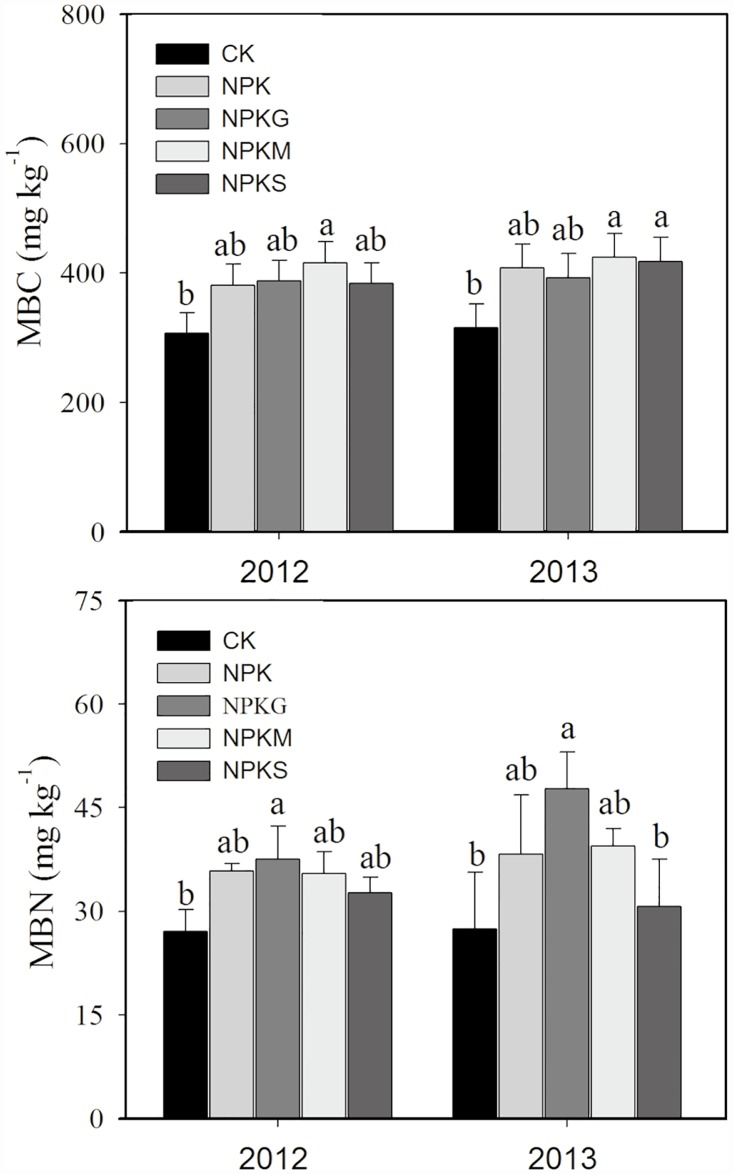
Variations in Microbial Biomass Carbon (MBC) and Nitrogen (MBN) under different fertilizations in 2012 and 2013. Vertical bars represent the SE (*n* = 3) and different letters above bars indicate significant differences between fertilizations at *P*<0.05.

The NPKM treatment exhibited significantly higher MBC than the CK over two crop seasons, whereas no significant difference was observed for MBC among the fertilized treatments, although comparatively higher MBC observations were recorded in NPKM-treated soil (Fig 2). Compared to the lowest MBN under CK, the NPKG treatment significantly increased MBN during the study period. Notably, the value of MBN under NPKS was equal to the other fertilized treatments in the 2012 late rice season, but was significantly lower than that of NPKG at the harvest of early rice season in 2013 (Fig 2).

**Fig 3:**
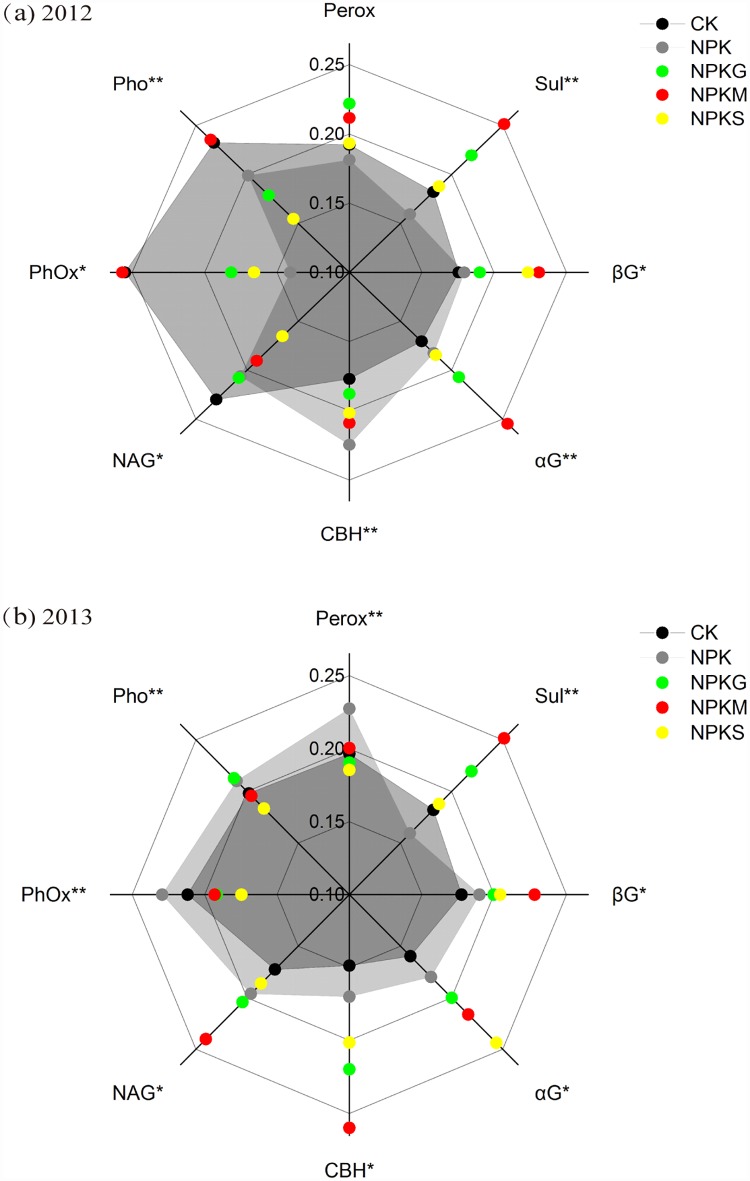
Radar graphs illustrating the relative responses of enzyme activity to different fertilizations in 2012 and 2013. To make a clear comparison when characterizing the organic amendments, the CK and NPK treatments were shown in two shadings. * and ** indicate significant differences between fertilizations at *P*<0.05 and *P*<0.01, respectively. Abbreviations: *αG*, α-glucosidase; *βG*, β-glucosidase; *CBH*, cellobiohydrolase; *NAG*, N-acetyl-glucosaminidase; *Perox*, peroxidase; *Pho*, phosphomonoesterase; *PhOx*, phenol oxidase; *Sul*, sulfatase.

Eight soil enzymes involved in C, N, P, and S cycling were identified in the second and third seasons of the trial (Fig 3). In the 2012 late rice season, all enzymes except peroxidase were strongly influenced by the experimental treatments. The activities of sulfatase, β-glucosidase, α-glucosidase and cellbiohydrolase were increased by 38.0% (12.70 μmol h^−1^ g^−1^ *vs*. 9.20 μmol h^−1^ g^−1^), 32.0% (169 μmol h^−1^ g^−1^ *vs*. 128 μmol h^−1^ g^−1^), 49.6% (55.8 μmol h^−1^ g^−1^ *vs*. 37.3 μmol h^−1^ g^−1^), and 12.8% (44.3 μmol h^−1^ g^−1^ *vs*. 37.5 μmol h^−1^ g^−1^), respectively, in the NPKM treatment compared with CK. By contrast, the NPKG and NPKS treatments had equal or lower enzyme activities compared with CK, except for phosphomonoesterase and N-acetyl-glucosaminidase. In the 2013 early rice season, the enzyme activity in the soils amended with NPK was generally similar to CK, with the exception of peroxidase. Most enzyme activities in the organic-treated soils were equal or higher than those of the NPK and CK treatments, whereas the activities of phenol oxidase and peroxidase were significantly lower compared with the NPK treatment.

**Fig 4:**
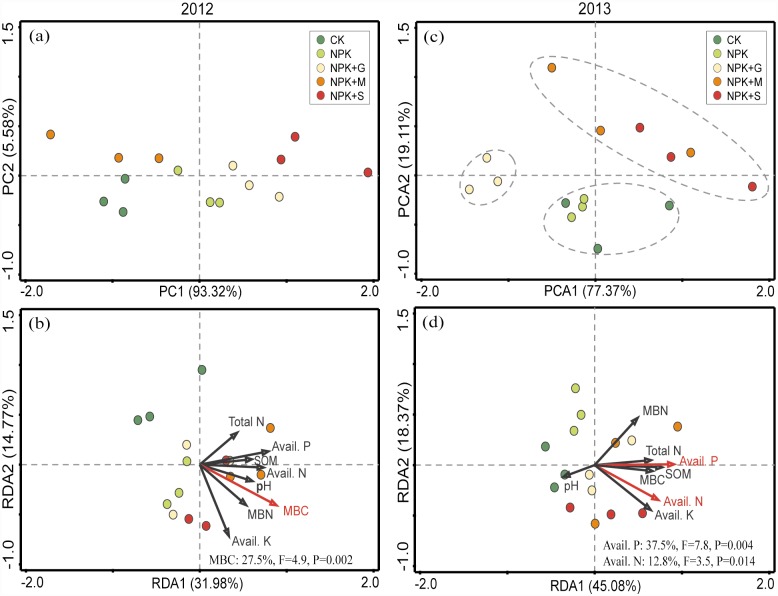
Principal Component Analysis (PCA) of enzyme activities in soils different fertilizations in the late rice season of 2012 (a) and the early rice season of 2013 (c), and Redundancy Analysis (RDA) results of the correlations between soil variables and enzyme activity in the late rice season of 2012 (b) and the early rice season of 2013 (d). The red arrows indicate the soil parameters that had a strong and significant impact on the enzyme activities (*P*<0.05), and the corresponding explained proportion of variability is shown in the lower right corner. Abbreviations: Abbreviations: *Avail*., available; *MBC*, microbial biomass carbon; *MBN*, microbial biomass nitrogen; *SOM*, soil organic matter.

Principal component analysis (PCA) showed that there was no obvious trend in soil enzyme activity in 2012 the late rice season (Fig 4a). Redundancy analysis (RDA) revealed that only the MBC significantly explained 27.5% of the total variability in enzyme activity (Fig 4b). In the 2013 early rice season of 2013, the tested treatments showed more pronounced differences in enzyme activities and were well separated into three distinct groups by the PC scores (Fig 4c). The RDA results indicated that AN and AP significantly affected the enzyme activities and explained 37.5% and 12.8%, respectively, of the total variability in enzyme activity (Fig 4d).

### Changes in the abundance and composition of microbial communities

**Fig 5:**
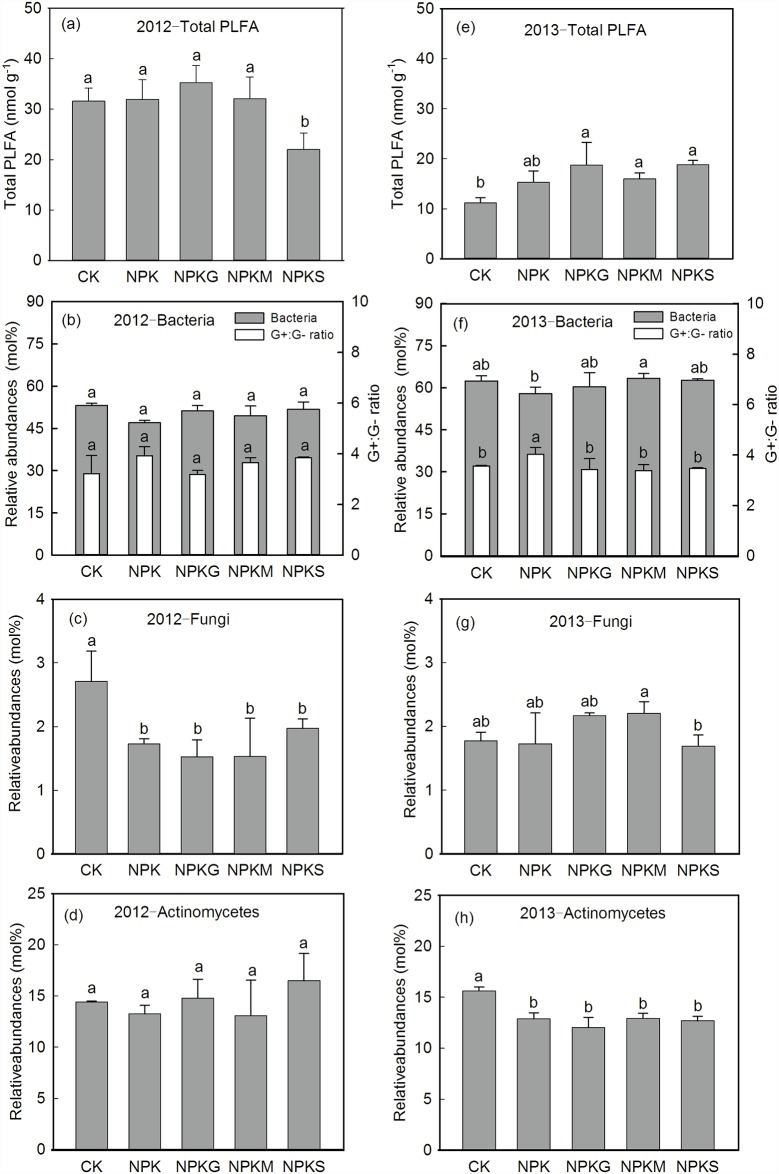
Variations of the total PLFA, relative abundance of bacteria, ratio of gram-positive to gram-negative bacteria, and relative abundances of fungi and actinomycetes under different fertilizations in the late rice season of 2012 and the early rice season of 2013. Vertical bars represent the SE (*n* = 3) and different letters above bars indicate significant differences between fertilizations at *P*<0.05.

The total PLFA ranged from 10.30 to 38.56 nmol g^−1^ (Fig 5). In the 2012 late rice season, there were no significant differences among the CK, NPK and NPKG and NPKM treatments, whereas their total PLFA contents were significantly higher than that of the NPKS treatment (Fig 5a). By contrast, in the 2013 early rice season the total PLFA contents were significantly increased by the organic amendments (NPKG, NPKM and NPKS), which increased the total PLFA contents by 67.0%, 42.2% and 67.7%, respectively, compared with the CK (Fig 5e). The NPK treatment had moderate levels of total PLFA and was not significantly different from CK and the organic amendments. In the 2012 late rice season, there were no significant differences between the tested treatments in the ratio of gram-positive to gram-negative bacteria and the relative abundances of bacteria and actinomycetes. However, fungal abundance was significantly decreased in the fertilized treatments compared with the CK, and the organic amendments did not affect fungal abundance beyond the fertilizer affect. In the spring of 2013 there was no effect of fertilization on fungi. In the 2013 early rice season, the NPK treatment had significantly higher ratios of gram-positive to gram-negative bacteria compared with the other treatments (Fig 5f). Actinomycete abundance showed a more pronounced response to the tested treatments and was significantly decreased in the fertilized treatments (NPK, NPKG, NPKM and NPKS), which reduced actinomycete abundances by 17.58%, 28.8%, 22.5% and 22.9%, respectively, compared with CK (Fig 5h). The organic additions also had no effect on actinomycetes with the fertilizer application.

**Fig 6:**
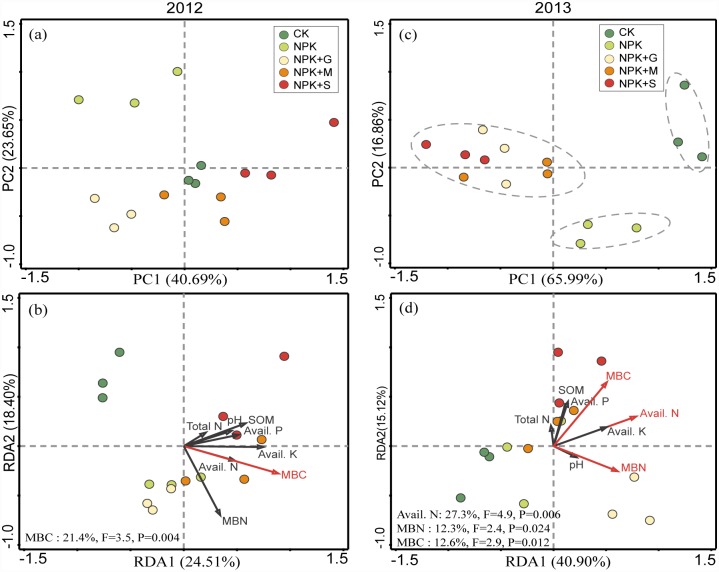
Principal Component Analysis (PCA) of microbial community composition (relative content of individual PLFA molecules) in soils under different fertilizations in the late rice season of 2012 (a) and the early rice season of 2013 (c), and Redundancy Analysis (RDA) of the correlations between soil parameters (chemical properties and microbial biomass C/N) and microbial community composition in the late rice season of 2012 (b) and the early rice season of 2013 (d). The red arrows indicate the soil parameters that had a strong and significant impact on the microbial community composition (*P*<0.05), and the corresponding explained proportion of variability is shown in the lower left corner. Abbreviations: *Avail*. available, *MBC* microbial biomass carbon, *MBN* microbial biomass nitrogen, *SOM* soil organic matter.

PCA analysis showed that the composition of the microbial community in the NPK-treated soil was significantly different from that of the other treatments in the 2012 late rice season (Fig 6a). This difference was related to the changes in the soil microbial biomass carbon (MBC), which explained 21.4% of the total variability in the composition of the microbial community, based on the RDA results (Fig 6b). Differences in microbial community composition among the treatments were more pronounced in the 2013 early rice season than in the 2012 late rice season (Fig 6c). The PLFA profiles of the CK-treated soil were well separated from those of the fertilized treatments along PC1, indicating that fertilization was a major factor affecting microbial community composition. A clear separation was also observed along PC2 when comparing the PLFA patterns of NPK treatment soil samples to the organic amendments (Fig 6d), implying secondary effects of organic inputs on the composition of soil microbial community. The RDA results showed that available N, MBC and MBN were significantly correlated with soil microbial composition and explained 27.3%, 12.6% and 12.3%, respectively, of the total community variability (Fig 6d).

### Rice yield response to organic amendment

As shown in Table 3, the fertilized treatments significantly improved rice yield compared to CK over the first 3 trial seasons. The significant rice yield increase observed in the first trial season suggested that fertilization is an effective management practice to improve crop production in yellow clayey soil. Although NPK treatment also significantly improved rice yield, the increase did not exceed the yields of the organic amendments. Based on the average yield of 3 crop seasons, the highest yield was obtained with the manure treatment (NPKM).

**Table 3:** Variations in rice yield under different fertilizations in 2012 and 2013.

Treatment	Rice yield (kg ha^−1^)			
	2012 (Early rice)	2012 (Late rice)	2013 (Early rice)	Mean
CK	3938±347c	5299±313c	3025±517c	4087±367d
NPK	5957±998b	6620±366b	6861±427b	6479±534c
NPKG	6455±436ab	7071±377ab	8012±168a	7179±190ab
NKPM	7188±59a	7357±168a	8567±322a	7704±144a
NPKS	6645±59ab	7088±448ab	6925±523b	6882±136bc
Two-way ANOVA results				
Treatment	*F* = 95.83	*P*<0.001		
Season	*F* = 11.36	*P*<0.001		
Treatment × Season	*F* = 8.37	*P*<0.001		

Data are means ± SE, *n* = 3. Different letters indicate significant differences between different fertilizations at *P*<0.05.

### Soil quality index

**Fig 7:**
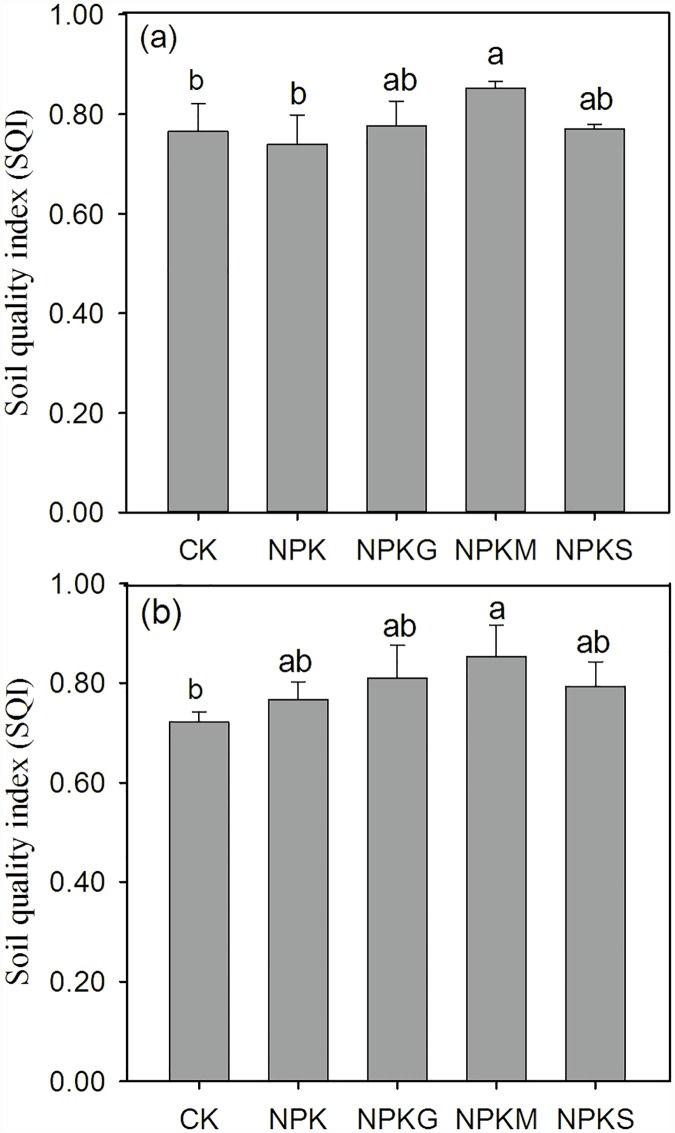
Variations in Soil Quality Index (SQI) under different fertilizations in the late rice season of 2012 (a) and the early rice season of 2013 (b).

After the TDS indicators were scored and weighted, the SQI was calculated using the Integrated Quality Index equation (Eq 1). SQI scores ranged from 0.70 to 0.92 for the tested treatments over the 2-studied crop seasons (Fig 7). The NPKM treatment received the highest SQI, and the opposite was true of CK. The changes in SQI in the remaining fertilized treatments were not statistically significant when compared to the CK treatment.

**Fig 8:**
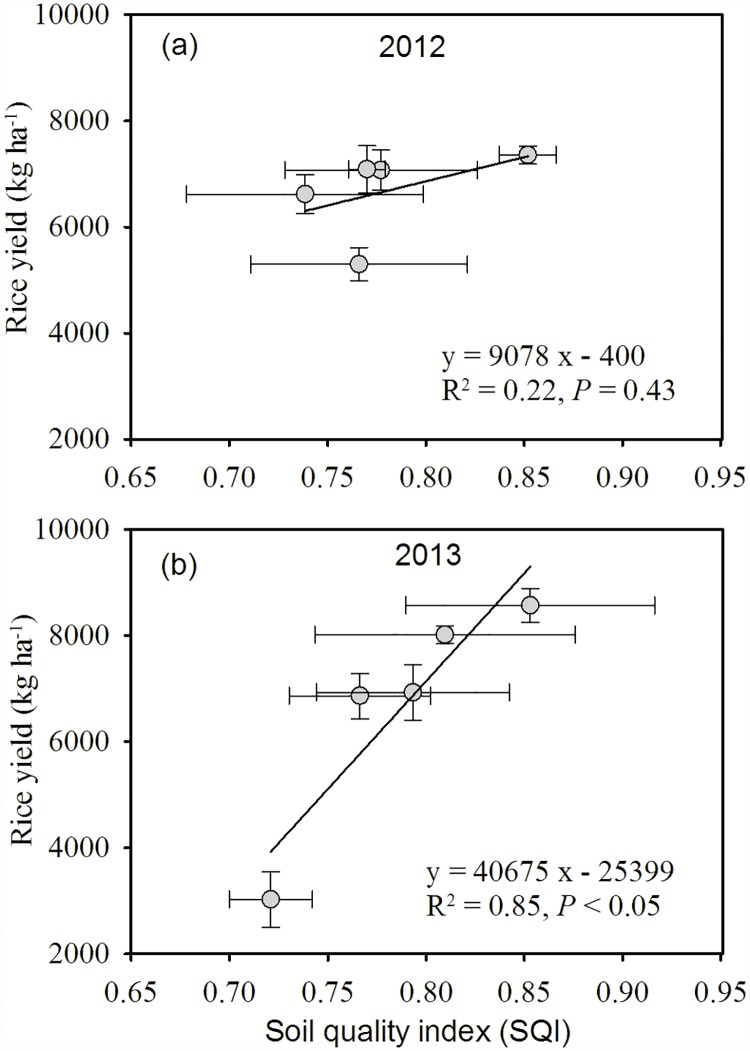
Relationships between Soil Quality Index (SQI) values and rice yields in 2012 and 2013.

In addition, the correlation between SQI and rice grain yield was significant (P < 0.05) in the 2013 early rice season, but not in the 2012 late rice season (Fig 8).

## Discussion

The results demonstrate significant changes in soil quality resulting from different fertilizer treatments (Fig 7). These changes with respect to productivity were most evident in the rice grain yield (Table 3). The NPKM treatment significantly increased rice yield, and was the highest SQI compared to other treatments. This is consistent with the view that organic materials can be an important input for increasing soil quality in rice production systems [30]. Monitoring the changes in soil quality indicators following a specific management strategy is a useful approach to determine the current soil quality status [23]. In this study, 22 soil variables including chemical and biological properties were measured to determine their responses to various inorganic and organic amendments.

Soil pH, TN and SOM showed no significant changes in response to the experimental treatments (Table 2), suggesting that changes in SOM, TN and pH were not been easily detected in the short term. Similar result was found by Chen *et al*. [38]. Differences among treatments occurred with respect to available N, P and K concentrations in the 2013 early rice season (Table 2). Organic amendments exhibited significantly higher AN and AK compared to the CK and NPK, which might be related to their additional inputs through the incorporation of organic materials. The soil AP concentrations significantly increased in the 2013 early rice season. This might be attributed to the warmer conditions (monthly mean air temperature, 29.0°C in July *vs* 18.6°C in October) at the time when the 2013 early rice season was harvested. Similar trend was observed by Bruun *et al*. [39]. The NPKM treatment showed the most favorable increase in P availability, which was significantly higher than the CK (Table 2). This is consistent with the finding of Hall *et al*. who attributed the increased P availability to the direct addition of P in the pig manure [40].

Soil microbial biomass is one of the most sensitive indicators of soil quality changes [2]. Significantly higher MBC, compared to CK, occurred only in NPKM during the 2012 late rice season and 2013 early rice season (Fig 2). This confirmed that farmyard manure, due to its high level of organic C, was more favorable for increasing soil organic C in a short term [41]. The organic amendments were incorporated at an equal rate of C. However, no significant differences were observed for SOC, compared with MBC. This supports the findings of Liu *et al*. [2], demonstrated that MBC was more sensitive and accurate than SOC for assessing soil quality. The RDA results showed that MBC was the key determinant affecting soil enzyme activities (Fig 4b). This suggests that MBC is an important active carbon source that serves as an energy supply and thus results in higher enzyme activity [42]. The NPKG treatment had the most positive effect on soil MBN during the two planting seasons (Fig 2). This might be related to a rapid turnover of green manure, which enables rapid N release [15].

Soil enzymes catalyze important reactions related to decomposition and nutrient turnover, and their activities can be used to measure soil health and as an early indicator of soil changes caused by agricultural practices [43]. We determined eight extracellular enzymes involved in C, N, P, and S cycling (Fig 3). Extracellular enzyme activities fluctuate frequently due to complex environmental conditions [44]. In contrast to the 2012 late rice season, significant changes in soil enzyme activities occurred in the early rice season in response to the treatments (Fig 3). In general, the organic amendments, especially under NPKM, were characterized as having relatively higher sulfatase, β-glucosidase, α-glucosidase, cellobiohydrolase, and N-acetyl-glucosaminidase activities, indicating improved soil health. Low phenol oxidase activity is conducive to the accumulation of soluble phenolics and thus inhibits the activity of hydrolytic enzymes [45]. In this way, both the NPKM and NPKS treatments may benefit soil C sequestration because of their relatively low phenol oxidase activity (Fig 3). In contrast, NPK might limit SOC content due to a high peroxidase activity under NPK, which negatively affects labile organic carbon fraction [46]. RDA showed that changes in enzyme activities depended on soil available P and N (Fig 4), consistent with the previous finding that soil enzymes are correlated with nutrient availability [47].

The soil microbial community responds strongly to both long-term land-use changes and short-term litter additions [48]. After a short period of plant residual addition to soil, net N immobilization and decreased inorganic N pools are often observed [49]. In this study, the straw was deficient in N negatively impacting the total PLFA in the 2012 late rice season (Fig 5a). A strong decrease in total PLFA occurred from October (2012 late rice harvest) to July (2013 early rice harvest), and the total PLFA content was significantly higher in the organic treatments in the 2013 early rice season. Similar finding was reported by Giacometti *et al*. [50]. Applying chemical NPK fertilizer significantly decreased the bacterial community compared with NPKM. This was also observed by Sun *et al*. [51]. The significantly higher ratio of G^+^/G^−^ observed in NPK (Fig 5f) was consistent with Billings and Ziegler [52] but different from results of Giacometti *et al*. [50]. Higher proportion of G^−^ bacteria usually occurs following a shift from oligotrophic to more copiotrophic conditions [53]. Addition of organic materials always results in a significant increase in G^−^ bacteria [54]. This might explain the relatively high ratio of G^+^/G^−^ in the NPK treatment (Table 2). External nutrients, including P, with fertilization supplied to soil, would result in significantly lower fungal abundance compared with the CK in the 2012 late rice season. Similar result was observed by Elzobair *et al*. [41]. Compared to the CK or NPK, no significant increases were observed for fungi in soils treated with organic amendments in the 2013 early rice season. Similar findings were reported by Giacometti *et al*. [50], who also found no significant differences of fungi in manure and crop residue treatments of a 50-year field experiment. In contrast, fungi abundance under NPKS was significantly lower than under NPKM (Fig 5g), suggesting that rice straw incorporation to paddy fields could negatively affect short-term soil microbial activity. This is probably due to its low MBN content (Fig 6). All fertilized treatments had decreased soil actinomycetes compared with the CK. This may be attributed to the enhanced acidity caused by N chemical fertilization, which is unfavorable for actinomycetes development [55].

Many studies have shown that soil microbial biomass and communities are changed by organic amendments and these changes may relate to soil C content [29]. Our results indicated that the PLFAs of the treatments were well separated by the PCA in the 2013 early rice season. The RDA results of the PLFAs confirmed that MBC was the dominant factor affecting the composition of the soil microbial communities in the yellow clayey soil (Fig 6). In addition, soil AN and MBN were the other two key determinants that affected the soil microbial community (Fig 6d). This is consistent with results of Cederlund *et al*. [56], who reported that soil N was the most important factor in the relative abundances of the studied microbial groups. Similar findings were also observed by Zhang et al. [30]. Pearson’s correlation analysis revealed that besides being correlated with most of the enzymatic activities total PLFAs content was also significantly correlated with MBN and MBC (S1 Table). This confirmed previous findings that soil biochemical and microbial properties were significantly correlated [50].

SQI is a primary indicator of sustainable land management [22]. Our results demonstrated that the highest SQI score was under NPKM (Fig 7). This indicated that chemical fertilization combined with manure might be a more sustained option for the development of yellow clayey soil. Fertilization practices can be controlled to modify soil fertility in a particular manner with time [57]. In this way, the non-significant correlation between SQI and rice yield in the 2012 late rice season (Fig 8a) was probably due to the relatively short experimental period, which caused slight changes in soil properties compared with those observed in the 2013 early rice season (Table 2 and Fig 3). The significant correlation between SQI and rice yield (Fig 8b) confirmed that SQI is a promising tool to integrate soil information and to indicate the soil quality degree of yellow clayey soil [2].

Based on these results, the application of chemical fertilizer plus manure to paddy fields is a useful way to enhance MBC, improve soil enzymes, increase the microbial community and thus improve soil quality and productivity in the yellow clayey soils of subtropical China.

## Conclusion

Fertilizer treatments significantly changed soil microbial biomass C and N, enzyme activities, microbial community composition, and rice yield after three crop seasons. Organic amendments showed more favorable impacts on nutrient availability and microbial activity and typically increased rice yield compared to the NPK treatment. The NPKM treatment was characterized by higher levels of available N, P and K, MBC, enzyme and microbial activities, and exhibited the highest SQI and rice yield. This indicated improved soil fertility and quality. Based on these results, application of chemical fertilizer plus manure is a practical option for enhancing soil labile C (e.g. MBC) and increasing the productivity of yellow clayey soil.

## Supporting information

S1 Fig Soil sampling procedure in each plot. (TIF)

S1 File Data for the cited Figs 1–8 in the manuscript. (XLSX)

S1 Table Pearson’s product-moment correlation coefficients among soil biochemical and microbial parameters. (DOCX)
