# Supplementary figures and images for: Effects of inorganic and organic amendment on soil chemical properties, enzyme activities, microbial community and soil quality in yellow clayey soil
Source: PLoS One. 2017 Mar 6;12(3):e0172767. doi: 10.1371/journal.pone.0172767 (PMC5338777; doi:10.1371/journal.pone.0172767)

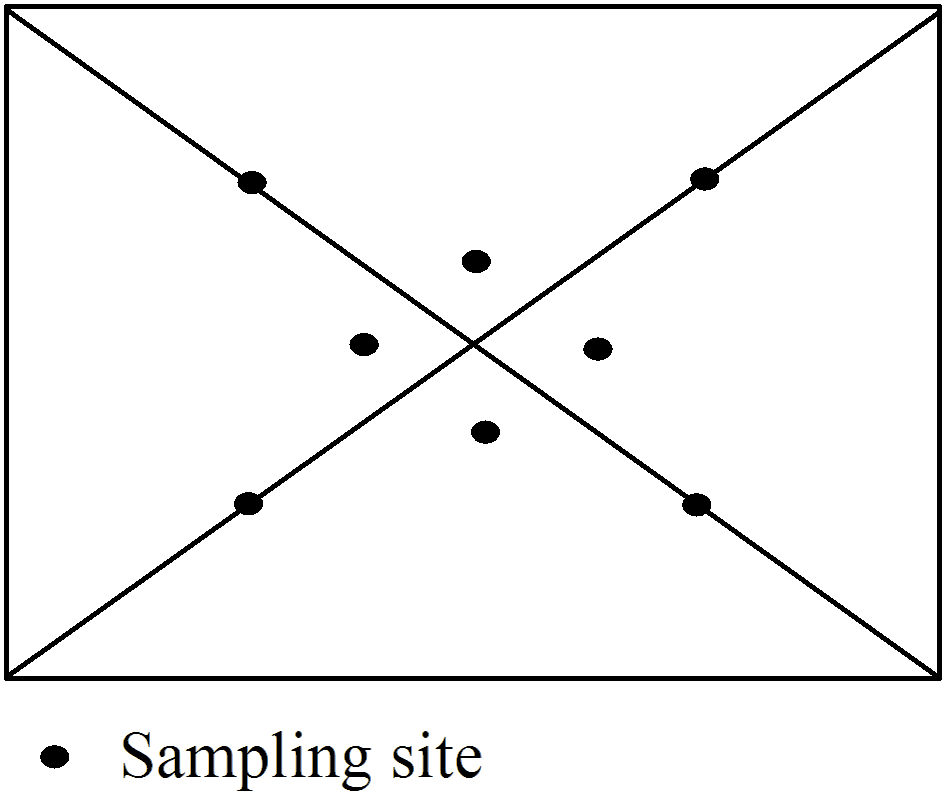

Supplement: S1 Fig — (TIF) [file pone.0172767.s001.tif]
