# Supplementary material for: Effects of inorganic and organic amendment on soil chemical properties, enzyme activities, microbial community and soil quality in yellow clayey soil
Source: PLoS One. 2017 Mar 6;12(3):e0172767. doi: 10.1371/journal.pone.0172767 (PMC5338777; doi:10.1371/journal.pone.0172767)
Supplement: S1 Table — (DOCX) [file pone.0172767.s003.docx]

**Table S1** Pearson’s product-moment correlation coefficients among soil biochemical and microbial parameters.

| Season | Soil variables | Pho | Sul | βG | CBH | NAG | αG | PhOx | Perox | MBC | MBN | PLFAs |
| --- | --- | --- | --- | --- | --- | --- | --- | --- | --- | --- | --- | --- |
| Late-rice season in 2012 | Pho | 1 |  |  |  |  |  |  |  |  |  |  |
|  | Sul | 0.329 | 1 |  |  |  |  |  |  |  |  |  |
|  | βG | 0.034 | **0.671**** | 1 |  |  |  |  |  |  |  |  |
|  | CBH | -0.094 | -0.092 | 0.255 | 1 |  |  |  |  |  |  |  |
|  | NAG | 0.424 | -0.021 | -0.301 | -0.302 | 1 |  |  |  |  |  |  |
|  | αG | 0.331 | **0.817**** | **0.557*** | 0.186 | -0.218 | 1 |  |  |  |  |  |
|  | PhOx | **0.632**** | 0.386 | 0.134 | -0.292 | 0.071 | 0.232 | 1 |  |  |  |  |
|  | Perox | 0.049 | **0.448*** | 0.209 | 0.114 | 0.046 | 0.262 | 0.297 | 1 |  |  |  |
|  | MBC | -0.092 | **0.624**** | **0.815**** | **0.511*** | -0.559 | **0.703**** | 0.038 | 0.254 | 1 |  |  |
|  | MBN | -0.162 | **0.451*** | 0.436 | 0.401 | -0.146 | **0.524*** | -0.392 | 0.372 | **0.564*** | 1 |  |
|  | Total PLFA | **0.453*** | 0.229 | -0.379 | -0.295 | 0.538* | 0.314 | 0.039 | 0.036 | -0.277 | 0.216 | 1 |
|  | Pho | 1 |  |  |  |  |  |  |  |  |  |  |
| Early-rice season in 2013 | Sul | 0.195 | 1 |  |  |  |  |  |  |  |  |  |
|  | βG | 0.186 | **0.823**** | 1 |  |  |  |  |  |  |  |  |
|  | CBH | 0.284 | **0.813**** | **0.851**** | 1 |  |  |  |  |  |  |  |
|  | NAG | 0.365 | **0.744**** | **0.813**** | **0.884**** | 1 |  |  |  |  |  |  |
|  | αG | 0.089 | **0.553*** | **0.761**** | **0.808**** | **0.566*** | 1 |  |  |  |  |  |
|  | PhOx | **0.671**** | -0.185 | -0.096 | -0.056 | 0.146 | -0.115 | 1 |  |  |  |  |
|  | Perox | **0.577*** | -0.202 | 0.042 | 0.081 | 0.262 | -0.037 | **0.771**** | 1 |  |  |  |
|  | MBC | 0.106 | **0.523*** | **0.851**** | **0.734**** | **0.699**** | **0.805**** | -0.056 | 0.172 | 1 |  |  |
|  | MBN | **0.661**** | **0.536*** | **0.591*** | **0.649**** | **0.734**** | 0.361 | 0.244 | 0.402 | **0.593*** | 1 |  |
|  | Total PLFA | 0.192 | 0.402 | **0.552*** | **0.673**** | **0.476*** | **0.771**** | -0.241 | -0.057 | **0.766**** | **0.562*** | 1 |

Abbreviations: *αG,* α-glucosidase; *βG,* β-glucosidase; *CBH,* cellobiohydrolase; *NAG,* N-acetyl-glucosaminidase; *Perox,* peroxidase; *Pho,* phosphomonoesterase; *PhOx,* phenol oxidase; *Sul,* sulfatase. * P≤0.05; ** P≤0.01.
